# Modifying the maker: Oxygenases target ribosome biology

**DOI:** 10.1080/21690731.2015.1009331

**Published:** 2015-02-03

**Authors:** Qinqin Zhuang, Tianshu Feng, Mathew L Coleman

**Affiliations:** 1 Tumour Oxygenase Group; School of Cancer Sciences; University of Birmingham; Birmingham, UK; 2 Centre for Cellular and Molecular Physiology; University of Oxford; Oxford, UK

**Keywords:** decoding, demethylase, hydroxylation, modification, oxygenase, ribosome, translation, 2-oxoglutarate

## Abstract

The complexity of the eukaryotic protein synthesis machinery is partly driven by extensive and diverse modifications to associated proteins and RNAs. These modifications can have important roles in regulating translation factor activity and ribosome biogenesis and function. Further investigation of ‘translational modifications’ is warranted considering the growing evidence implicating protein synthesis as a critical point of gene expression control that is commonly deregulated in disease. New evidence suggests that translation is a major new target for oxidative modifications, specifically hydroxylations and demethylations, which generally are catalyzed by a family of emerging oxygenase enzymes that act at the interface of nutrient availability and metabolism. This review summarizes what is currently known about the role or these enzymes in targeting rRNA synthesis, protein translation and associated cellular processes.

## Introduction

Ribosome biogenesis and protein synthesis are highly orchestrated and dynamically regulated cellular processes that are tightly controlled by the modification of key regulatory factors. Modification of chromatin at rDNA loci controls rRNA production,^1^ the rate limiting step of ribosome biogenesis. rRNA is itself heavily modified by base and ribose methylation and pseudouridylation, which together likely promote rRNA stability and translation efficiency.^2^ Ribosomal proteins and translation factors are modified by phosphorylation, methylation, hypusination, dipthamide modification, and others types of modification.^3-7^ Such a complex array of diverse modifications have likely evolved to optimize ribosome biogenesis and translational efficiency, to promote heterogeneity in ribosome populations destined for alternative tasks,^8^ and to allow fine control of protein synthesis rate in response to nutrient availability and stress.^9^

Growing interest in a family of oxygenases that catalyze diverse oxidative modifications to DNA, RNA and protein has led to the recent discovery that the cellular machinery controlling rRNA and protein synthesis and protein translation are the target of hydroxylation and demethylation. This review aims to introduce the family of oxygenase enzymes thought to be predominantly responsible for such modifications and to summarize what is currently known about the role of oxygenases in protein synthesis.

## 2-Oxoglutarate-oxygenases

**Figure 1.**
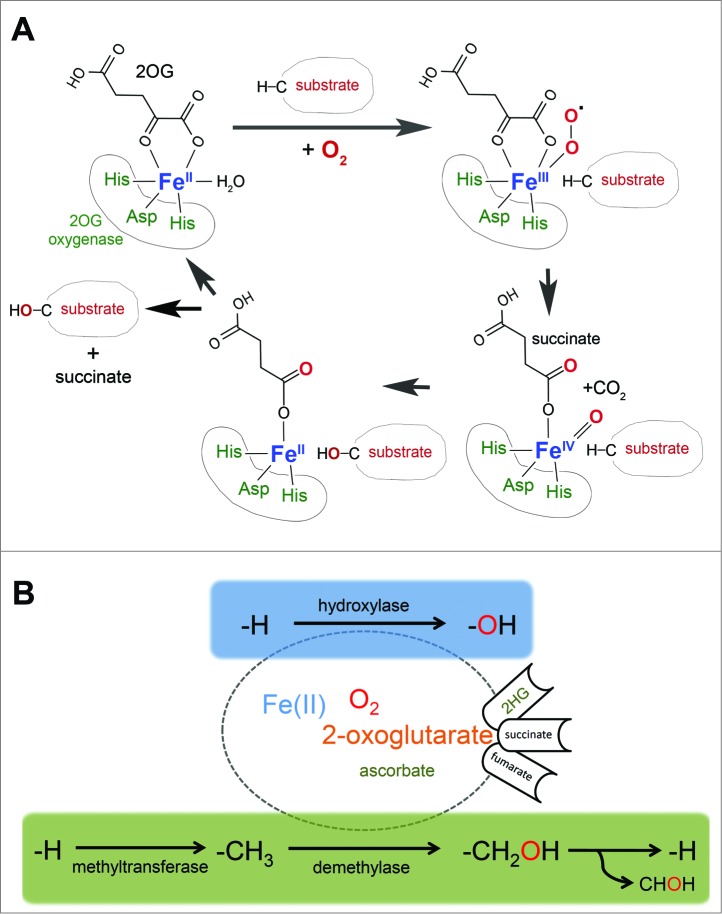
2-oxoglutarate-oxygenase catalysis.(**A**) Catalytic cycle. Catalysis requires essential co-factors Fe(II), molecular oxygen (O_2_), and the Krebs cycle intermediate 2-oxoglutarate (2OG), together with the ‘2-His 1-carboxylate’ motif (His-Asp-His) within the active site of the enzyme. Note that one atom of oxygen from molecular oxygen is incorporated into the product, and that the reaction generates succinate and carbon dioxide. (**B**) 2OG-oxygenases catalyze stable hydroxylation (blue box) and demethylation *via* hydroxylation (green box) of DNA, RNA, lipid and protein. Note that ascorbate is required for full activity of a subset of 2OG-oxygenases (hence the smaller font). Hydroxylation of a methyl group generally creates a highly unstable hydroxymethyl intermediate that decomposes into formaldehyde (CHOH) and the unmodified residue. The fate of the formaldehyde is not known, but may be metabolized by formaldehyde dehydrogenase. Note that in some chemical contexts hydroxylation of a methyl group can create a stable hydroxymethyl product, such as that catalyzed by TETs. The oncometabolite 2-hydroxyglutarate (2HG) can interfere with 2OG-oxygenase function by acting as an activating co-substrate in some instances, or as a 2OG competitive inhibitor in others. Succinate and fumarate inhibit 2OG-oxygenases by product inhibition and 2OG competition, respectively.

Oxygenases whose activities depend on Fe(II), oxygen, and the Krebs cycle intermediate 2-oxoglutarate (2OG) (‘2OG-oxygenases’) form a family of relatively poorly characterized enzymes consisting of more than 60 members in mammals.^10^ 2OG-oxygenases catalyze site-specific modifications, with specificity being driven by primary and secondary sequence constraints in the substrate and structural determinants within the enzyme. The catalytic domain of 2OG-oxygenases consists of a ‘double-stranded β helix’ (DSBH), a structural arrangement that has evolved to present specific amino acid side chains within the active site to optimally co-ordinate co-factors and substrate.^11^ A ‘facial triad' of amino acids belonging to the conserved HXD/E…H motif orchestrates iron coordination (**Fig. 1A**). In the presence of molecular oxygen, oxidative decarboxylation of 2OG releases succinate and carbon dioxide and generates a highly reactive Fe(IV)-oxo intermediate that drives hydroxylation of the prime substrate^12^ (**Fig. 1A**). In eukaryotes, currently described modifications catalyzed *via* this mechanism are thus far limited to hydroxylation, and demethylation catalyzed *via* a hydroxylation reaction.^10^ Hydroxylation of a methyl group generally results in the formation of a highly labile hydroxymethyl intermediate that rapidly decomposes releasing formaldehyde with consequent reversal of the methyl modification (**Fig. 1B**). 2OG-oxygenases catalyzing demethylation include the JmjC histone demethylases, important epigenetic modifiers widely implicated in development, physiology and disease.^13^

Possibly the most well-known examples of stable hydroxylation catalyzed by 2OG-oxygenases are prolyl and lysyl modification of the extracellular matrix protein collagen,^14^ together with the role of hydroxylation in hypoxia signaling mediated by the Hypoxia Inducible transcription Factor (HIF).^15^ In the latter example, three hydroxylases (PHD1-3) modify two conserved prolyl residues in the HIFα subunit that targets it for rapid proteasomal destruction. The activities of HIFα prolyl hydroxylases are compromised under conditions in which the availability of the essential co-factor oxygen is limited (hypoxia), leading to HIFα protein stabilization.^15^ Thus, a relatively low affinity for molecular oxygen imparts an oxygen sensing role on the HIFα hydroxylases, allowing HIFα stabilization and activation in hypoxia to drive transcriptional programs that have evolved to bring about adaption to this important physiological and pathological stress.^15^

The role of the HIF hydroxylases in regulating transcription has highlighted the potential for gene expression control by 2OG oxygenases. Indeed, it has since become apparent that these enzymes target the cellular machinery governing gene expression at multiple levels. For example, several 2OG oxygenases with nucleotide hydroxylase activity have now been identified, including the ALKBH family (see below) and the TET family that hydroxylate and demethylate 5-methylcytosine.^16^ The JmjC histone demethylases mediate both transcriptional activation and repression at the level of chromatin.^10,13^ JMJD6 catalyzes 5-lysyl hydroxylation of mRNA splicing factor U2AF65, and modulates mRNA splicing.^17-20^ This review will focus on recent literature describing protein synthesis as a major new target of 2OG-oxygenases.

## 2OG-oxygenases Target Protein Synthesis

Emerging evidence indicates that in addition to their role in controlling gene expression at the stages outlined above, 2OG oxygenases are also involved in translational control *via* modification of rDNA loci, RNAs, ribosomal proteins and translation factors.

### Histone Demethylases Regulate rRNA Transcription

**Figure 2.**
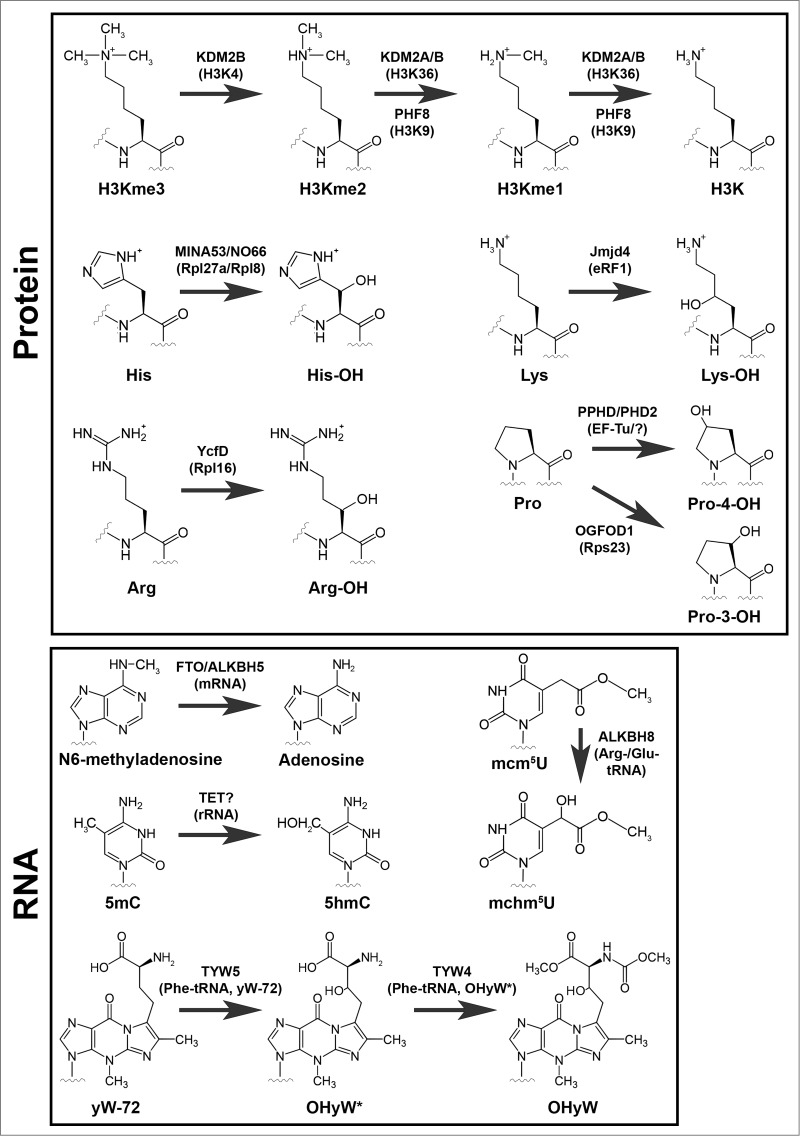
Modifications catalyzed by 2OG-oxygenases in ribosome biology. 2OG-oxygenases are in bold above the arrows, with the corresponding substrate in brackets underneath. **Upper panel:** Modifications to protein. The modifications presented in the top row represent histone lysine demethylation by various JmjC histone demethylases. H3K=Histone H3 lysine. me=methyl group. The second and third rows in this panel represent stable hydroxylation of amino acid side chains by the indicated 2OG-oxygenases. **Lower panel:** Modifications to RNA. Presented in the same format as the upper panel. 5mC=5-methylcytosine. 5hmC=5-hydroxymethylcytosine. mcm^5^U=methoxycarbonylmethyluridine. mchm^5^U=methoxycarbonylhydroxymethyluridine. yW=wybutosine. yW-72=wybutosine minus 72Da. OHyW*=undermodified hydroxywybutosine. OHyW=hydroxywybutosine, formed by attachment of methyl and methoxycarbonyl groups to the aminocarboxyl side chain of OHyW* by TYW4.

**Figure 3.**
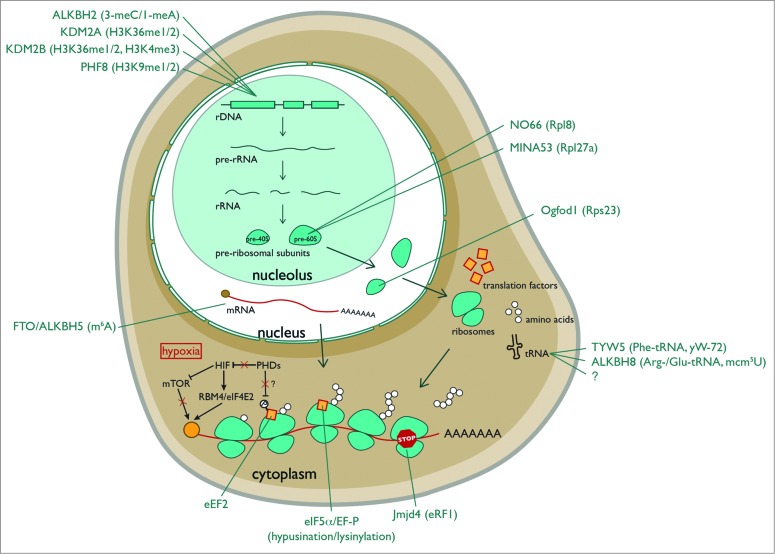
Hydroxylation and demethylation events in eukaryotic ribosome biogenesis and protein translation. ALKBH2 is a demethylase that repairs alkylated rDNA. 3-meC=3-methylcytosine. 1-meA=1methyladenine. KDM2A/B and PHF8 are nucleolar histone lysine demethylases that target rDNA. MINA53 and NO66 are nucleolar histidyl hydroxylases of the large ribosomal subunit. Ogfod1 is a nuclear prolyl hydroxylase of the small ribosomal subunit. FTO and ALKBH5 are m^6^A RNA demethylases. TYW5 and ALKBH8 hydroxylate the anti-codon loop of the indicated tRNAs. The ‘?’ under ALKBH8 denotes an as yet unidentified mcm^5^U hydroxylase. Jmjd4 is a hydroxylase of the translational termination factor eRF1. Note that hypoxia (red box) substantially regulates translation. Inhibition of prolyl hydroxylases in hypoxia indirectly represses EIF4E (*via* HIF-dependent mTOR inhibition) while activating the translation of specific transcripts *via* an RBM4/HIF2α/eIF4E2 cap-dependent mechanism. The orange ball represents the cap.

rDNA transcription is under the control of several chromatin modifiers, including members of the 2OG-oxygenase family.^1^ KDM2A is a mono and dimethyl histone H3 lysine 36 (H3K36me1/2) demethylase (**Figs. 2 and 3**) localized to the nucleolus where it binds to the rDNA promoter and represses rDNA transcription.^21^ KDM2B is a nucleolar H3K4Me3 demethylase (**Figs. 2 and 3**) that represses rDNA transcription and cell growth and suppresses tumorigenesis^22^ (**Table 1**). In contrast, PHF8 is a H3K9me1/2 demethylase (**Fig. 2 and 3**) that binds to the promoter region of rDNA to promote rDNA transcription.^23^ Thus, the earliest step in ribosome biogenesis and protein synthesis is under the control of opposing histone demethylases of the 2OG-oxygenase family. An intriguing possibility is that the JmjC demethylases could also act at later stages of ribosome biogenesis. For example, ribosomes and chromatin both consist of charged nucleic acids in complex with small basic proteins that are often rich in lysine and arginine. Similar to histones, ribosomal proteins are subject to a range of methylations including arginine and lysine. Therefore, it is possible that nucleolar JmjC demethylases could target methylated ribosomal proteins in addition to histones. Consistent with this speculation, non-histone targets have been identified for JmjC histone demethylases.^24^

**Table 1.** 2OG-oxygenases with targets in ribosome biology and protein synthesis are frequently implicated in disease, particularly cancer. It should be noted that other substrates of these enzymes may exist in other biological contexts and that the critical targets of these enzymes involved in disease are often unclear, but may include the translational targets listed. The role of JmjC family 2OG-oxygenases in disease was recently reviewed by Oppermann and colleagues^10^

Translational Oxygenase	Translation target	Diseases
KDM2A	rDNA promoter (H3K36me1/2)	cancer
KDM2B	rDNA promoter (K3K4me3 and H3K36me1/2)	cancer
PHF8	rDNA promoter (H3K9me1/2)	cancer, mental retardation
ALKBH2	rDNA 1-meA and 3-meC	cancer
ALKBH5	mRNA N6-methyladenosine	obesity
FTO	mRNA N6-methyladenosine	obesity, cancer, alzheimer's, cardiovascular
TET1-3	rRNA 5-methylcytosine	cancer, neurodegeneration
ALKBH8	Arg-/Glu-tRNA (mcm5U)	—
TYW5	Phe-tRNA (yW-72)	—
MINA53	Rpl27a	cancer, asthma, autoimmunity
NO66	Rpl8	cancer
OGFOD1	Rps23	–
Jmjd4	eRF1	cancer

### Nucleotide Oxygenases

AlkB is a highly conserved 2OG oxygenase in *Escherichia coli* that removes methylation adducts in DNA using a hydroxylation mechanism.^25,26^ It has 8 human homologues, termed ALKBH1-8. Of these, 3 have currently been implicated in targeting the protein synthesis machinery. ALKBH2 promotes rDNA transcription by repairing alkylation damage associated with rapid transcription (**Fig. 3**).^27^ ALKBH5 demethylates N6-methyladenosine (m^6^A) (**Figs. 2 and 3**), one of the most prevalent nucleotide modifications in mRNA and long noncoding RNA.^28,29^ N6-methyladenosine is recognized by specific RNA-binding proteins that modulate RNA stability, and mediates widespread gene regulation.^30,31^ The function of ALKBH5 m^6^A-demethylation may be related to nuclear RNA export, perhaps consistent with its nuclear localization.^29^ Loss of ALKBH5 is associated with defective spermatogenesis in mice, consistent with its enriched expression in the testes.^29^ Interestingly, m^6^A is also a target of the 2OG-oxygenase FTO.^28^ A common variant in the FTO gene was originally identified as a risk factor for increased BMI and predisposition to obesity^32^ (**Table 1**). Gene knockout studies suggest that FTO targets a specific subset of m^6^A-containing mRNAs.^33^ FTO may target other methylated nucleotides under specific conditions, although the function of these modifications is not yet known.^28,34^ Since FTO is primarily expressed in the brain, and ALKBH5 in the testes, tissue-specific expression of these enzymes may avoid functional redundancy.

The TET family of 2OG-oxygenases (TET1-3) mediate epigenetic DNA modification by converting 5-methylcytosine (5mC) to 5-hydroxymethylcytosine (5hmC).^16,35^ Subsequent, oxidation to 5-formylcytosine (5fC) and 5-carboxylcytosine can also bring about full reversal of the methylation. These cytosine modifications are thought to mediate their biological effects by modulating DNA duplex stability and DNA-protein (transcription factor) interactions,^16^ which in turn may in part explain the role of this subfamily of 2OG-oxygenases in cancer and neurodegeneration^36^ (**Table 1**). However, it is possible that other targets of TET enzymes could also be involved. Of interest here is the fact that 5mC, 5hmC and 5fC are described in databases of RNA modifications.^37^ Furthermore, recent evidence suggests that TET enzymes can indeed catalyze the formation of 5hmC in RNA^38^ (**Fig. 2**), raising the possibility that this class of 2OG-oxygenases could also be novel regulators of protein synthesis.

The anticodon stem and loop region of tRNA is subject to a variety of modifications that optimize tRNA folding, prevent frameshifting and ensure accurate codon selection.^39^ For example, the uridine at position 34 at the wobble position of the anticodon loop in specific tRNAs is modified to 5-methoxycarbonylmethyluridine (mcm^5^U).^40^ This modification is mediated by the action of the methyltransferase domain of ALKBH8 on 5-carboxymethyluridine (cm^5^U).^41,42^ Interestingly, ALKBH8 is a bifunctional enzyme that also encodes a 2OG-oxygenase domain which hydroxylates mcm^5^U to 5-methoxycarbonylhydroxymethyluridine (mchm^5^U)^43,44^ (**Figs. 2 and 3**). Through its action on tRNA modification ALKBH8 is proposed to improve decoding of non-cognate codons^44^ and to enhance the translation of proteins enriched in arginine and glutamic acid residues, including key DNA damage response proteins.^41,45^ Indeed, loss of ALKBH8 confers sensitivity to DNA damage.^41,45^ However, the importance of the 2OG-oxygenase domain in this pathway is unclear, and will require structure-functional analyses involving complementation of ALKBH8 null cells with hydroxylase defective mutants. Recently described transgenic mice with ‘knock-in’ alleles of either methyltransferase- or oxygenase-mutant ALKBH8 will allow such studies.^44^ Interestingly, this mouse model identified that mchm^5^U exists in 2 diastereomers and that only one of these is catalyzed by ALKBH8. Therefore, an independent hydroxylase also targets mcm^5^U, perhaps a different member of the ALKBH1-8 family.

The gaunosine at position 37 of phenylalanine tRNA is modified to a tricyclic base with a bulky side chain known as Wybutosine.^46^ Similar to the cm^5^U modifications described above, Wybutosine suppresses frame-shifting and maintains translational fidelity.^47^ Wybutosine derivatives include hydroxywybutosine, formation of which is catalyzed by the 2OG-oxygenase TYW5^48^ (**Figs. 2 and 3**). However, the function of hydroxywybutosine is currently unclear. Knockdown of TYW5 did not confer a gross phenotype in HeLa cells, suggesting this modification does not play a critical role in bulk translation or cell growth, at least under normal growth conditions.^48^ It is interesting to speculate that TYW5 may be required for the efficient translation of phenylalanine-rich proteins involved in specific biological process (e.g. nucleoporins), akin to the role of ALKBH8 in the DNA damage response.

### Ribosomal Oxygenases

MINA53 is a 2OG-oxygenase that was recently assigned as a histidyl hydroxylase of the 60S large subunit protein Rpl27a^49^ (**Fig. 2 and 3**). MINA53 was first identified in a microarray screen for novel Myc target genes, and was subsequently shown to be required for tumor cell proliferation.^50^ MINA53 is overexpressed in some tumors relative to normal tissues, and high level of MINA53 expression may be associated with poor patient prognosis in some contexts^51-55^ (**Table 1**). However, other studies have reported that MINA53 overexpression is associated with favorable prognosis in early stages of lung cancer.^52^ Therefore, the exact role of MINA53 in tumorigenesis remains unclear, but could be highly context specific. MINA53 has also been independently studied in other contexts in addition to cancer, including allergy^56^ and immunity, where it appears to regulate T-cell differentiation^57,58^ (**Table 1**). However, in both cases the molecular mechanisms involved remain unclear. Although MINA53 was reported to be a demethylase of H3K9me3,^59^ detailed biochemical and structural analyses have raised questions about its biochemical activity.^49,60^ More recently, unbiased proteomics coupled to *in vitro* peptide screening identified Rpl27a as a *bona fide* substrate of MINA53.^49^ Rpl27a is hydroxylated in a MINA53-dependent manner on a specific histidine residue at position 39 within a HHH motif (where the hydroxylated residue is underlined) (**Fig. 2**). Endogenous Rpl27a purified from human and mouse cell lines, normal mouse and human tissues, and tumors is hydroxylated to near completion.^49^ The abundance of the modification may argue against a signaling role and may be more consistent with a structural function. The modified residue is located on a disordered loop that extends into the core of the ribosome. However, higher resolution ribosomal structures and functional studies are required to pinpoint the role of the HHH motif and its hydroxylation in Rpl27a function. Interestingly, Rpl27a H39 is adjacent to the residue implicated in cycloheximide binding and sensitivity in yeast,^61^ although these effects were not manifest in mammalian cells defective for MINA53 (Dr Adam Zayer, personal communication). Furthermore, MINA53 knockout mice are viable and fertile,^56^ suggesting that Rpl27a hydroxylation is not essential for normal development and reproduction.

NO66 is closely related to MINA53, sharing 39% sequence homology overall and 57% homology within the catalytic domain.^62,63^ Like MINA53, NO66 is also found in the nucleolus^63^ and is implicated in cancer cell growth, particularly in non-small cell lung carcinoma^64^ (**Table 1**). Proteomic screens coupled to *in vitro* peptide screening identified the 60S large ribosomal subunit Rpl8 as a NO66 substrate^49^ (**Figs. 2 and 3**). Consistent with the sequence conservation with MINA53, NO66 is also a histidyl hydroxylase, modifying Rpl8 at position 216 within a motif similar to Rpl27a (HQH) (**Fig. 2**). Endogenous Rpl8 purified from human and mouse cell lines, normal mouse and human tissues, and tumors is hydroxylated to near completion.^49^ Similar to Rpl27a, the hydroxylated residue is within a disordered loop that extends into the ribosome, but in this case it is proximal to the peptidyl-transferase center (PTC), perhaps suggestive of an important role in translation. Mutation of the corresponding residue in yeast Rpl8 (Rpl2) affects peptidyl-tRNA binding, PTC activity and confers resistance to the antibiotic sparsomycin.^65^ However, chronic NO66 knockdown in human cells does not appear to drastically affect polysome profiles, cell growth or sparsomycin sensitivity in our hands (Tianshu Feng, personal communication). This disparity could reflect the difference between non-conservative mutation and a relatively subtle modification. However, it does suggest that PTC activity is unlikely to be grossly affected by Rpl8 hydroxylation. Perhaps NO66 activity and Rpl8 hydroxylation regulate the translation of specific mRNA's or simply fine tune the structural integrity of the ribosome in the vicinity of the PTC.

In prokaryotes, a 2OG oxygenase named YcFD that is highly related to NO66 catalyzes argininyl hydroxylation of the 60S ribosomal protein L16 at position 81^49^ (**Fig. 2**). Structural and phylogenetic analyses indicate that NO66 was likely evolved from YcFD, and MINA53 from NO66 in a much later gene duplication event.^49,60^ Similar to MINA53 and NO66, the function of Rpl16 hydroxylation is unknown. Rpl16 R81 hydroxylation is essentially complete in wildtype strains and absent in YcFD gene knockouts.^49^ Surprisingly, both knockout and overexpression of YcFD are associated with reduced growth potential under some circumstances.^49,66^

In addition to NO66 and MINA53, a third eukaryotic ribosomal protein hydroxylase was recently identified. The 2OG-oxygenase OGFOD1 is distantly related to the HIF prolyl hydroxylases, and catalyzes hydroxylation of an evolutionary conserved prolyl residue in Rps23, a component of the 40S small subunit^67,68^ (**Figs. 2 and 3**). Interestingly, Rps23 is doubly hydroxylated by the OGFOD1 ortholog in yeast and algae,^69^ but only singly hydroxylated in higher eukaryotes.^67,68^ Similar to Rpl27a and Rpl8, Rps23 hydroxylation is essentially complete in all cells and tissues. The target prolyl residue, corresponding to Pro62 in humans, is located at the apex of a loop that projects into the decoding center of the ribosome, which led to the postulation that hydroxylation is required for optimal translational accuracy.^68,69^ Using reporters of stop codon decoding as a measure of translational accuracy it was shown that the inhibition of OGFOD1 orthologs has variable effects on stop codon readthrough.^68,69^ OGFOD1 inhibition modestly enhances translational termination in human and *Drosophila* cells,^67,68^ whereas more dramatic effects were observed in yeast in a bidirectional manner that was highly context-specific.^69^ Despite observing no measurable loss in translational termination efficiency, inhibition of OGFOD1 in *Drosophila* and human cells is often associated with marked translational arrest phenotypes including: reduced protein synthesis; increased eIF2α phosphorylation; stress granule formation and autophagy.^67,68^ Therefore, the role of altered translational termination in the phenotypes reported was questioned. It would be of interest to investigate whether other measures of translational accuracy and decoding might explain the growth deficits observed. Conversely, the deletion of OGFOD1 ortholog Tpa1 in yeast resulted in substantial changes in translational termination.^69^ Although associated growth alterations were not reported, Tpa1 knockout cells are viable, which suggests that levels of endogenous stop codon readthrough were compatible with growth in this context.

Further investigation is required to determine whether deregulated ribosomal hydroxylase activity drives diseases associated with these enzymes (such as cancer), or explains the complex phenotypes associated with enzyme ablation in eukaryotes. Some 2OG-oxygenases have multiple substrates, raising the possibility that other targets may also exist that contribute to the role of these enzymes in physiology and disease.

## Translation Factor Hydroxylases

The first example of a hydroxylated translation factor was discovered in the context of hypusine, a uniquely modified amino acid only found in eIF5α^70^ (**Fig. 3**). Hypusine is formed by the transfer of an n-butylamine group from spermidine to the lysyl side chain, followed by hydroxylation.^70,71^ In this case hydroxylation is catalyzed by a unique enzyme that is structurally distinct to 2OG-oxygenases. Deoxyhypuysine hydroxylase is a HEAT-repeat-containing dinuclear iron enzyme that catalyzes the final step in hypusine formation in an oxygen- and Fe(II)-dependent,^72,73^ but 2OG-independent,^74^ manner.

Consistent with a fundamental role in protein synthesis, eIF5α has been shown to promote elongation and the translation of polyproline motifs.^75,76^ Importantly, the hypusine modification of eIF5α is essential for its function,^75-78^ perhaps related to the proximity of the modification to the acceptor stem of the P-site tRNA.^75^ The importance of eIF5α in protein synthesis and eukaryotic development and viability is underscored by its evolutionary conservation, with a homolog also present in bacteria (EF-P). Interestingly, the lysine residue that is modified to hypusine in eIF5α is conserved in EF-P, where it is also subject to an unusual modification.^79^ Lysine 34 is post-translationally modified by a β-lysine residue. Importantly, EF-P and eIF5α modifications share additional similarities. Following lysinylation, lysine 34 is modified by a hydroxylase termed YfcM,^80^ which is distinct from the eIF5α HEAT-repeat metalloenzyme and structurally unrelated to 2OG-oxygenases.^81^ Similar to eIF5α, EF-P has been implicated in elongation and translation of polyproline tracts.^82,83^

Recent evidence suggests that the regulation of elongation factors by hydroxylation extends beyond EF-P/eIF5α. Structure-directed bioinformatics analyses identified a 2OG-oxygenase in *Pseudomonas* related to the HIF prolyl hydroxylases, which was subsequently shown to target the EF-Tu elongation factor^84^ (**Fig. 2**). EF-Tu delivers aminoacyl-tRNA to the ribosome and releases tRNA in response to codon recognition.^85^ Although the *Pseudomonas* PHD hydroxylates the Switch I loop of the EF-Tu GTPase domain,^84^ no change in GTP hydrolysis was observed following hydroxylation. Consistent with this, no difference in global translation rate was detected in a *Pseudomonas* strain with a mutation in the EF-Tu hydroxylase. Perhaps hydroxylation regulates other aspects of EF-Tu functions in translation. Interestingly, EF-Tu hydroxylase mutant *Pseudomonas* strains exhibit reduced growth in the presence of iron chelators,^84^ which may be consistent with a role of this 2OG-oxygenase in iron sensing and regulation.

EF-Tu and its structural homolog EF-G share almost identical binding sites on the ribosome and act sequentially in the elongation cycle. EF-G is the evolutionary ancestor of eukaryotic eEF2, an essential factor for eukaryotic protein synthesis due to its role in promoting the GTP-dependent translocation of the ribosome.^86^ As such, eEF2 activity is subject to tight regulation, including by phosphorylation at threonine 56 within its GTPase domain.^87^ The eEF2 Kinase is activated in response to cellular stress such as nutrient and oxygen starvation by AMP Kinase.^87^ Interestingly, PHD2, the oxygen sensing HIF hydroxylase (see above), is implicated in a pathway that regulates eEF2 T56 phosphorylation in response to acute hypoxia (**Figs. 2 and 3**). PHD2 siRNA and 2OG-oxygenase inhibition with a 2OG competitor both mimicked acute hypoxia by inducing eEF2 T56 phosphorylation.^88^ Although the authors of this study speculate that eEF2 could be a novel PHD2 substrate, PHD2 appears not to hydroxylate peptide sequences derived from the Switch I loop,^84^ and is therefore unlikely to be analogous to the EF-Tu system in *Pseudomonas*. Further investigation is required to unravel the role of PHD2 in regulating signaling to eEF2 phosphorylation.

Taken together, the work outlined thus far in this section implicates translational elongation factors as highly conserved targets of structurally distinct hydroxylase enzymes. However, a recent study also implicates translational termination as a target of 2OG-oxygenases.^89^ Jmjd4 is a 2OG-oxygenase whose catalytic domain is most similar to the splicing factor lysyl hydroxylase Jmjd6. In contrast to Jmjd6, Jmjd4 is predominantly expressed in the cytoplasm, suggesting an alternative function. Indeed, proteomic pulldowns did not identify splicing factors, and instead identified the eukaryotic translational termination factor eRF1 as a specific interactor that bound to Jmjd4 in an activity-dependent manner.^89^ eRF1 is responsible for decoding the stop codon in the A-site of the ribosome. Together with the GTPase eRF3A, eRF1 stimulates peptidyl-tRNA hydrolysis to release the mature polypeptide.^90^ Mass spectrometry (MS) sequencing of endogenous eRF1 identified the hydroxylation of K63 (**Figs. 2 and 3**), which was dependent on Jmjd4 activity. Similar to ribosomal protein hydroxylation, quantitative MS suggested that eRF1 K63 hydroxylation is essentially complete in the steady state.^89^ Furthermore, the fact that eRF1 K63 hydroxylation was highly conserved and ubiquitous across multiple tissues and cultured cell lines suggests a fundamental role in eRF1 function. K63 is located within a highly conserved NIKS motif within a domain of eRF1 implicated in stop codon decoding and its coupling to peptidyl-tRNA hydrolysis (the N-domain, or domain-1).^91-95^ Interestingly, cross-linking experiments suggest that K63 contacts the invariant uridine in stop codons.^96^ Consistent with a direct role for the NIKS motif in stop codon recognition, a recent structure of the eukaryotic ribosome in complex with eRF1/eRF3A localized the NIKS sequence in direct proximity of the stop codon.^97^ Importantly, inhibiting Jmjd4 activity increases stop codon readthrough, as measured using a bicistronic Renilla/STOP/Firefly reporter vector.^89^ Reduced translational termination efficiency was observed in response to loss of Jmjd4 activity in a range of tissue culture cells and with a variety of stop codon contexts^89^ (Tianshu Feng, personal communication). Importantly, comparing the *in vitro* release factor activity of recombinant wildtype or K63R mutant eRF1 exposed to Jmjd4 allowed the effects of Jmjd4 activity *in vivo* to be specifically localized to eRF1 K63 hydroxylation.^89^

## Decoding: A Key Target of 2OG-oxygenases in Translation?

Although the hydroxylation and demethylation events reviewed here have been discovered across ribosome biology, common roles may be emerging in elongation and decoding. With respect to decoding, 3 hydroxylation events described above are directly linked to translational fidelity; (i) Rps23 proline 62 hydroxylation (OGFOD1), (ii) anti-codon loop hydroxylation of tRNAs (TYW5, ALKBH8), and (iii) N-domain hydroxylation of eRF1 (Jmjd4). It is of interest to highlight the latter 2 examples, where independent hydroxylases target the codon reading domains of tRNA and a peptidyl tRNA mimic. Thus, at least within the limits of the studies published to date, 2 hydroxylations may be present in the decoding center of the ribosome at any one time, with hydroxylated Rps23 proline 62 in the proximity of eRF1 K63 hydroxylation or tRNA hydroxylated at wybutosine (TYW5) or mcm^5^U (ALKBH8). Interestingly, an obligate partner of the methyltransferase activity of ALKBH8 (Trm112)^42^ is also required for the activity of a methyltransferase that targets eRF1,^98^ raising the possibility that there may be cross-talk between eRF1 and tRNA modifications. Considering that an unidentified tRNA mcm^5^U exists^44^ (see above), and many members of the 2OG-oxygenase family remain poorly or completely uncharacterised,^10^ it is possible that other examples of hydroxylation targeting decoding may emerge.

Decoding is considered to be a major determinant of biological ‘fitness’ and as such is a highly evolved process.^99,100^ Decoding requires fast and accurate selection of the correct tRNA from a pool of competitors and involves conformational changes to both the ribosome and the tRNA (or its mimic). Perhaps oxygenases have allowed evolution to fine tune the architecture of the ribosome using hydroxylation, a relatively subtle modification, which could optimize protein-protein and protein-RNA interactions, efficient codon recognition and/or conformational rearrangements. Alternatively, the oxidative modifications described here may allow decoding to sense nutrient availability *via* oxygenase activity. Interestingly, reduced translational fidelity can be advantageous under some circumstances. For example, decoding errors can promote adaption in response to stress in bacteria.^101^ Perhaps a collective reduction in decoding center hydroxylation under conditions of stress (e.g., amino acid starvation, metabolic flux, and/or hypoxia) could reduce translational fidelity to signal adaptive responses. In such a scenario, the oxygenases would be acting as sensors relaying changes in nutrient availability and metabolism to ribosomal decoding.

## Translational Oxygenases as Sensors

The cofactor requirements of 2OG-oxygenases (2OG, Fe(II), O_2_) place them at a unique interface between nutrient availability and metabolism (**Fig. 1**). Enzymes with a relatively low affinity for one or more co-factors have the potential to act as sensors of that nutrient (and its metabolic predecessors). The clearest example of a sensing role thus far is for the hypoxia-responsive HIF system, as outlined above. Indeed, hypoxia is well-known to have multiple profound effects on translation control^9^ (**Fig. 3**). There is a substantial decrease in eIF4E-driven cap-dependent translation in hypoxia, at least partly due to mTOR inhibition *via* HIF-dependent and -independent pathways.^102,103^ Translation of proteins involved in the adaptive response to hypoxia is maintained *via* IRES control^9^ and a novel eIF4E2-mediated cap-dependent mechanism that involves direct binding of HIF2α^104^. Therefore, HIF hydroxylases may contribute to translational control indirectly *via* HIF regulation, and perhaps more directly *via* alternative substrates (such as those regulating eEF2 phosphorylation).

HIF-independent but oxygen-sensitive mechanisms of translational control could be of interest with respect to the wider family of 2OG-oxygenases. Examples of translational oxygenases acting as oxygen sensors are currently lacking however. Although MINA53, NO66, OGFOD1 and Jmjd4 all require oxygen for hydroxylation of their respective substrates they can maintain efficient catalysis under conditions of severe hypoxia.^49,68,69,89^ Whether other enzymes discussed here (e.g. RNA hydroxylases/demethylases) could be more sensitive to oxygen starvation is not yet known. For those enzymes that have been tested and found to be relatively insensitive to hypoxia it is possible that a concurrent reduction in one or more other co-factors could cause a more dramatic loss in activity. It is interesting to note that the EF-Tu prolyl hydroxylase of *Pseudomonas* may have a relatively high K_m_ for Fe(II),^84^ raising the possibility that this and perhaps other translational hydroxylases may sense Fe(II), and thereby link Fe(II) availability to translational control. Furthermore, some 2OG-oxygenases are competitively inhibited by intermediates of the TCA cycle such as fumarate and succinate (‘oncometabolites’, **Fig. 1B**), which are elevated in diseases associated with fumarate hydratase and succinate dehydrogenase deficiency, respectively.^105,106^ Neomorphic mutations in isocitrate dehydrogenases lead to 2-hydroxyglutarate production (**Fig. 1B**) in glioblastomas and acute myeloid leukemia cancers, which is associated with variable effects on the activity of some 2OG-oxygenases.^105-108^ It is possible that translational oxygenases might also communicate these metabolic disturbances to gene expression control at the level of protein synthesis. Such disturbances in co-factor availability and metabolism could cause a significant reduction in a specific hydroxylation or demethylation event within translation, or perhaps more modest effects on multiple modifications that collectively modulate protein synthesis.

Since many of the oxidative modifications described here go to near completion (e.g., ribosomal proteins and eRF1), how quickly would a reduction in 2OG-oxygenase activity due to reduced co-factor availability lead to a loss of the modification and a biological response? Reversal of some post-translational modifications can lead to rapid loss of the modification in the absence of the forward reaction. However, although theoretically feasible, a reversal enzyme for hydroxylation has yet to be described. Isotopic labeling and mass spectrometry experiments indicated that hydroxylation catalyzed by a HIF asparaginyl hydroxylase is unlikely to be reversed.^109^ Therefore, a reduction in hydroxylation following co-factor depletion would rely on natural turnover of the substrate, with relatively stable substrates only eliciting altered biological responses following chronic nutrient depletion (such as those found in pathological conditions for example).

## Future Perspectives

Taken together, the literature reviewed here supports protein synthesis as a major new target of 2OG-oxygenases. Since many of these enzymes remain uncharacterized, and those that have been studied have the potential to target multiple substrates, it seems likely that the list of translational oxygenases will continue to grow. These may also be complemented by novel classes of hydroxylases as exemplified by the eIF5α HEAT-repeat hydroxylase and the YfcM hydroxylase of EF-P. Although current examples of hydroxylases targeting the translational machinery appear to be enriched within elongation and decoding, it is possible that oxygenases will also be discovered that target other key regulatory steps such as initiation and recycling.

2OG-oxygenases are commonly deregulated in disease^10^ (see also Table 1), likely due to their action at the interface of nutrient availability and metabolism, and their common role in gene expression and growth control. These enzymes have small druggable active sites that are amenable to small molecule inhibition.^110^ As such, 2OG-oxygenases are attracting significant interest as novel therapeutic targets. Considering the role of protein synthesis in disease, further work characterizing the role of 2OG-oxygenases in translation and their potential as drug targets is warranted. This should include attempts to clarify the function of those modifications reviewed here where the physiological significance remains unclear, together with efforts aimed at discovering novel translational oxygenases.
